# Advances of Targeted Therapy in Treatment of Unresectable Metastatic Colorectal Cancer

**DOI:** 10.1155/2016/7590245

**Published:** 2016-04-05

**Authors:** Suk-young Lee, Sang Cheul Oh

**Affiliations:** Division of Oncology/Hematology, Department of Internal Medicine, College of Medicine, Korea University, 148 Gurodong-ro, Guro-gu, Seoul 08308, Republic of Korea

## Abstract

Despite being one of the most frequently diagnosed cancers worldwide, prognosis of metastatic colorectal cancer (CRC) was poor. Development and introduction of biologic agents in treatment of patients with metastatic CRC have brought improved outcomes. Monoclonal antibodies directing epidermal growth factor receptors and vascular endothelial growth factor are main biologic agents currently used in treatment of metastatic CRC. Encouraged by results from many clinical trials demonstrating efficacy of those monoclonal antibodies, the combination therapy with those targeted agents and conventional chemotherapeutic agents has been established as the standard therapy for patients with metastatic CRC. However, emergency of resistance to those target agents has limited the efficacy of treatment, and strategies to overcome the resistance are now being investigated by newly developed biological techniques clarifying how to acquire resistance. Here, we introduce mechanisms of action of the biologic agents currently used for treatment of metastatic CRC and several landmark historical clinical studies which have changed the main stream of treatment. The mechanism of resistance to those agents, one of serious problems in treatment metastatic CRC, and ongoing clinical trials to overcome the limitations and improve treatment outcomes will also be presented in this review.

## 1. Introduction

Colorectal cancer (CRC) is the fourth most commonly diagnosed cancer and the third leading cause of disease mortality in the United States [1]. Approximately 20% of patients with CRC present with distant metastasis at the time of diagnosis [2]. Additional 25–35% develops metastasis metachronously during the disease course [3]. Prognosis of patients with metastatic CRC was dismal in the past with the median overall survival (OS) of about 8 to 12 months when fluorouracil and leucovorin were the only therapeutic options [4]. Introduction of monoclonal antibodies, such as antiepidermal growth factor receptor (EGFR) antibody or antivascular endothelial growth factor (VEGF) antibody, in combination with the chemotherapeutic agents in treatment of metastatic CRC have brought improvement of survival, and recent clinical trials performed with those monoclonal antibodies at first-line treatment showed median survival of 17.9 to 29.9 months [5–7]. Encouraged by these results, anti-EGFR or anti-VEGF antibodies are now recommended as the standard therapy of first-line chemotherapy in treatment of metastatic CRC. This review is focused on targeted therapies applicable to patients with unresectable metastatic CRC, mechanisms of action of the biologic agents, and limitations of the targeted therapies and solutions.

## 2. EGFR-Targeted Therapies

**Figure 1:**
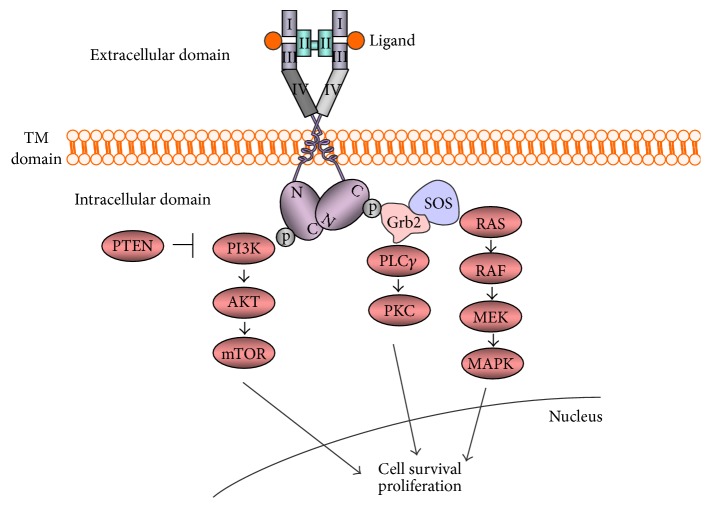
Signaling through EGFR. Signaling is initiated by interaction of ligands with EGFR. The resultant autophosphorylation of tyrosine kinase residues binds to the growth-factor-receptor-bound protein 2 (GRB2), and SOS is recruited to the plasma membrane. Subsequent activation of RAS activates RAS-RAF-MEK-MAPKs pathway. PI3Ks-AKT or RAS-PLC*ε*-PKC are also known to be activated by signaling through EGFR. TM: transmembrane.

The ERBB family of receptors consist of 4 members, EGFR and EGFR-related receptors (HER2, HER3, and HER4). EGFR, a receptor tyrosine kinase (RTK), is ubiquitously expressed in epithelial, mesenchymal, and neuronal cells and play a role in development, proliferation, and differentiation [8]. The ERBB family of RTKs are transmembrane receptors consisting of an extracellular domain, a single hydrophobic transmembrane segment, and an intracellular domain containing a preserved tyrosine kinase residue [9]. The signaling through the EGFR is initiated with binding of ligands to domains I and III of extracellular domain, the binding site of the receptor. The binding of ligands induces formation of heterodimer or homodimer between the receptor family members leading to autophosphorylation of tyrosine kinase residue in the carboxy-terminus of the receptor protein. The autophosphorylated receptors subsequently activate downstream intracellular signaling pathways such as RAS-RAF-mitogen-activated protein kinase kinase- (MEK-) mitogen-activated protein kinase (MAPKs), or phosphatidylinositol 3-kinase- (PI3K-) AKT pathway. Other than these pathways, phospholipase C- (PLC*γ*-) protein serine/threonine kinase C (PKC) pathway is also known to be activated by EGFR [10–13] (Figure 1).

### 2.1. Cetuximab and Panitumumab

Cetuximab and panitumumab are monoclonal antibodies targeting EGFR and block activation of downstream signaling pathways. Cetuximab is a chimeric monoclonal antibody, whereas panitumumab is a fully humanized monoclonal antibody [14]. A preclinical study using the xenograft model of human colorectal carcinoma was performed to determine the potential therapeutic utility of the cetuximab when combined with CTP-11 [15]. The study showed synergistic activity of cetuximab with CTP-11 in inhibiting growth in a series of cell lines even in CTP-11 refractory cell lines.

For induction chemotherapy to convert unresectable metastatic disease to resectable status, several randomized controlled trials were performed to access efficacy of cetuximab combined with chemotherapy. The CELIM randomized controlled phase II trial assigned patients with nonresectable liver metastases to receive cetuximab with FOLFOX6 or FOLFIRI. Overall response rate (ORR) was not significantly different between two groups (odds ratio (OR), 1.62, 0.74–3.59; *p* = 0.23). Retrospective analysis of response rate by* KRAS* mutational status resulted in 70% of a partial or complete response in* KRAS* wild-type cancers; meanwhile, there was 41% of ORR in cancers with* KRAS* mutation (OR 3.42, 1.35–8.66; *p* = 0.008). Resectability changed from 32% to 60% after chemotherapy in patients with wild-type* KRAS* (*p* < 0.0001) [16]. Another randomized controlled trial compared cetuximab plus chemotherapy (FOLFIRI or mFOLFOX6) to chemotherapy without the targeted agent in patients with unresectable liver metastases from CRC harboring wild-type* KRAS*. Significantly different* R*0 resection rate was observed between two groups with 25.7% in cetuximab plus chemotherapy groups and 7.4% in chemotherapy only group (*p* < 0.01) [17]. A meta-analysis of four randomized controlled trials analyzing resectability in patients with wild-type* KRAS* CRC whose metastatic lesions are limited in the liver reported that the addition of cetuximab or panitumumab to chemotherapy significantly increased the* R*0 resection rate from 11% to 18% (relative risk (RR), 1.59; *p* = 0.04) and ORR (RR, 1.67; *p* = 0.0001) comparing to chemotherapy alone [18].

Therefore, to increase the resectability of liver metastasis, cetuximab combination with chemotherapy could be selected.

As expected, benefit of anti-EGFR monoclonal antibodies was evaluated initially in patients with postprogression metastatic CRC. The BOND study, the first study demonstrating the clinical utility of cetuximab with convincing evidence, was performed in 329 patients with CRC who experienced disease progression on treatment with irinotecan-based regimen. Results of this large phase III study comparing cetuximab with or without irinotecan showed significant improvement of ORR and median PFS in irinotecan plus cetuximab group comparing with cetuximab monotherapy group (ORR 23% versus 11%; *p* = 0.007, time to progression 4.1 versus 1.5 months; *p* < 0.001). No difference in OS was observed, but patients with mutant* KRAS* were included in this study [19]. A single-agent cetuximab was also examined for its efficacy in patients with CRC previously exposed to chemotherapeutic agents. Cetuximab was revealed to improve OS (hazard ratio (HR), 0.77; 95% confidence interval (CI), 0.64–0.92; *p* = 0.005) and PFS (HR, 0.68; 95% CI, 0.57–0.80; *p* < 0.001) comparing with the best supportive care in this study [20]. Another phase III trial compared the efficacy of cetuximab plus irinotecan with irinotecan monotherapy in patients with CRC who experienced progression to first-line therapy with fluoropyrimidine and oxaliplatin. The study failed to improve OS (HR, 0.975; 95% CI, 0.854–1.114; *p* = 0.71), the primary endpoint of this study. In this study, patients with immunohistochemical expression of EGFR were enrolled regardless of mutational status of* RAS* [21]. Panitumumab has also been studied as a single agent or in combination with FOLFIRI in patients with CRC exposed to first-line chemotherapy. Patients with wild* KRAS* exon 2 tumors were proven to benefit from treatment with panitumumab in terms of improved PFS [22–25]. On the other hand, panitumumab failed to meet the primary endpoint of improved OS in randomized multicenter PICCOLO trial, in which the efficacy of panitumumab plus irinotecan was compared with irinotecan alone in patients with wild-type* KRAS* tumors resistant to fluoropyrimidine treatment with or without oxaliplatin (HR, 1.01; 95% CI, 0.83–1.23; *p* = 0.91). Inclusion of patients with* NRAS* or* BRAF* mutation in this study might have been one of causes for the failure considering the result that patients with any mutation among* KRAS*,* NRAS*, or* BRAF* who received panitumumab plus irinotecan showed detrimental effect in OS in this study [26] (Table 1).

Efficacy of both anti-EGFR monoclonal antibodies was also examined in first-line treatment of patients with CRC (Table 2). In the CRYSTAL trial, patients were randomly assigned to receive FOLFIRI or FOLFIRI plus cetuximab as first-line therapy. The significant improvement of PFS was proven in patients harboring wild-type* KRAS* exon 2 (9.9 months versus 8.7 months; HR, 0.68; 95% CI, 0.50–0.94; *p* = 0.02) who received cetuximab plus FOLFIRI [6, 27]. The recently updated data proved the significant benefit in PFS again as well as OS (23.5 versus 20.0 months; HR, 0.796; *p* = 0.0093) with the addition of cetuximab in the combination chemotherapy in* KRAS* exon 2 wild-type patients [6]. Outcomes comparing efficacy of FOLFOX with or without cetuximab were also reported. The retrospective analysis of patients with known* KRAS* exon 2 mutational status registered in the randomized phase II OPUS trial showed significantly better ORR (61% versus 37%; odds ratio, 2.54; *p* = 0.011) in patients treated with cetuximab in combination with FOLFOX. Statistically significant improvement of PFS was also demonstrated in wild-type* KRAS* exon 2 population receiving cetuximab plus FOLFOX, but the difference was only 15 days (7.7 versus 7.2 months; HR, 0.57; 95% CI, 0.36–0.91; *p* = 0.016) [28]. However, a randomized phase III MRC COIN trial reported no significant benefit of cetuximab combined chemotherapy (FOLFOX or capecitabine/oxaliplatin) in terms of OS (17.9 versus 17.0 months; *p* = 0.67) or PFS (8.6 versus 8.6 months; *p* = 0.60) in patients with locally advanced or metastatic CRC harboring wild-type* KRAS* exon 2 [29]. In addition to this study, no benefit of PFS or OS was also reported in the randomized phase III NORDIC VII study which investigated the efficacy of cetuximab in combination with oxaliplatin-containing regimens in patients with advanced or metastatic CRC as first-line therapy [30]. The common finding in COIN and NORDIC VII study is that infusional fluorouracil (FU) was not used in these studies suggesting combination of chemotherapeutic agent and the targeted agent might be important to affect outcomes. Modality of administration is another factor to consider regardless of the addition of cetuximab given the cell-cycle specific cytotoxic effect of FU. Meanwhile, recently reported results from the randomized phase III CALGB/SWOG 80405 trial showed effectiveness of FOLFOX combined with cetuximab in first-line treatment [32]. The optimal combination with chemotherapy and a targeted agent should be confirmed with further clinical trials. Treatment with panitumumab plus either FOLFOX or FOLFIRI has been studied in patients with metastatic CRC. Results of the open-label, randomized PRIME trial investigating efficacy of FOLFOX with or without panitumumab as the first-line treatment in patients with all* RAS* wild-type CRC showed significant improvement of PFS (HR, 0.72; 95% CI, 0.58–0.90; *p* = 0.004) and OS (HR, 0.77; 95% CI, 0.64–0.94; *p* = 0.009) in those treated with the combination of panitumumab and FOLFOX [31, 33].

### 2.2. Significance of* KRAS*,* NRAS*, and* BRAF* Mutation Status

It has been reported that overexpression of EGFR is observed in 49% to 82% of CRC [34–37]. Since EGFR is the target of therapy with anti-EGFR monoclonal antibodies, it is easily expected that its expression level could be a possible predictive factor for outcomes of treatment with agents directing the receptor. However, contrary to the expectation, it has been known that assessment of EGFR expression status with immunohistochemistry (IHC) is not helpful in the prediction of treatment efficacy. It was reported that a 25% ORR was achieved in CRC without expression of EGFR by IHC [38]. Other several data also showed no correlation of EGFR expression intensity of colorectal tumor cells with response rate to the anti-EGFR therapy. In addition, the low treatment efficacy of anti EGFR monoclonal antibodies in patients with CRC was reported, and these outcomes highlighted the necessity of investigation on the potential predictive markers for response to cetuximab [19, 39, 40].

In light of the fact that the RAS-RAF-MEK-MAPKs pathway is the downstream signaling cascade for the EGFR, mutations of molecular components of this pathway have been evaluated as the predictive markers for the anti-EGFR therapy. Investigation into the molecular basis was based on the retrospective analyses using tumor tissue of patients who participated in clinical trials. Mutations in codons 12 and 13 of exon 2 of* KRAS* gene resulting in constitutive activation of the downstream signaling cascade have been demonstrated to be insensitive to treatment with anti-EGFR monoclonal antibodies, cetuximab, and panitumumab [24, 27, 28, 41, 42]. The benefit of the use of anti-EGFR monoclonal antibodies in patients with wild-type* KRAS* was proven in both treatment with single-agent of cetuximab or panitumumab and that with combination chemotherapy plus those monoclonal antibodies [24, 27, 28, 41]. Patients with CRC harboring mutant* KRAS* gene have been excluded from the use of cetuximab or panitumumab based on those results.

Activating mutations in* RAS* other than the* KRAS* exon 2 mutation have also been studied to answer the heterogenous clinical response in terms of poor response to the EGFR-directed therapy in patients with CRC harboring the wild* KRAS* exon 2. It has been turned out that additional mutations with resultant constitutive KRAS activation can occur at exon 3 (codons 59 and 61) and exon 4 (codons 117 and 146) of* KRAS* or* NRAS* gene, another member of the* RAS* oncogene family, through the sequencing studies, although more than 80% of* KRAS* mutations are found at codons 12 and 13 [43–45]. A previous study which investigated the frequency of* KRAS*,* NRAS*, and* BRAF* mutations in CRC reported that mutations of the* NRAS* at codons 12, 13, and 61 range from approximately 3% to 5% [46]. The controversial role of those infrequent* RAS* mutations beyond the* KRAS* exon 2 mutations has been recently clarified in several studies. A study which analyzed patients from PRIME trial reported that 17% of 641 patients originally categorized as not having* KRAS* exon 2 mutations revealed having mutations in exons 3 and 4 in* KRAS* or exons 2, 3, and 4 in* NRAS* gene. The study demonstrated no benefit of treatment with panitumumab combined with FOLFOX in patients harboring* KRAS* or* NARAS* mutations and even deteriorated effect in these patients [33]. A recently published FIRE-3 study also suggested detrimental effect of all* RAS* mutations on outcomes of treatment with cetuximab plus FOLFIRI in patients with tumors harboring* RAS* mutations by showing significantly worse PFS than that of patients with* RAS* mutations treated with bevacizumab, an agent inhibiting angiogenesis, plus FOLFORI [7].

Despite the clarified mechanism of the lack of response of colorectal tumors with mutated* RAS* gene to the EGFR-directed therapy, certain tumors having wild-type* RAS* gene are known not to respond to that therapy. Although* BRAF* has been considered one of the candidate molecules responsible for the resistance for its role as a downstream effector of RAS, its usefulness as a predictive marker has not been determined. A V600E mutation in* BRAF* gene is found in about 5% to 9% of CRC [47]. The planned subgroup analysis with patients from the PRIME trial suggested the correlation of* BRAF* mutation with poor prognosis but failed to demonstrate its role as a predictive marker to the therapy with panitumumab combined with FOFOX [33]. The* BRAF* gene as a prognostic factor has also been suggested in an updated analysis of the CRYSTAL trial by showing worse prognosis in patients with* BRAF* mutation than in patients with wild type [6]. In addition,* BRAF* gene mutation status was also prognostic for OS in patients with CRC treated with capecitabine with or without bevacizumab [48]. A recent report of systematic review and meta-analysis of 21 studies indicated high-risk clinicopathologic characteristics in colorectal tumors with* BRAF* mutations in terms of TMN stage (T4 tumors), differentiation (poor differentiation), and tumor location (proximal location) [49].

On the other hand, a randomized phase II COIN trial indicated that cetuximab may have a detrimental effect in patients with CRC harboring* BRAF* mutation treated with capecitabine and oxaliplatin or FOLFOX as the first-line chemotherapy [29]. Several retrospective studies also suggested the role of* BRAF* as a marker of resistance to the EGFR-directed therapy in patients with metastatic CRC who experienced progression on the first-line therapy [50–53]. Furthermore, recent prospective data from the PICCOLO trial consistently reported the dismal effect of panitumumab combined with irinotecan on patient with* BRAF* mutations in the subsequent lines setting of chemotherapy [54]. Based on these results,* BRAF* mutation is now suggested as a prognostic factor in patients with CRC, and current guideline recommends genotyping of the gene at diagnosis of stage IV disease. And we suggest that biomarkers for targeted agents should be developed at the early phase trials.

## 3. Antiangiogenesis Therapy

Vascular endothelial growth factors (VEGFs) are a large family of growth factors involved in physiologic and pathologic angiogenesis. The family is composed of 5 members, VEGF-A, VEGF-B, VEGF-C, VEGF-D, and placental growth factor (PLGF) [55]. The proangiogenic effect of VEGFs is exerted by binding to their receptors consisting of VEGFR-1 (Flt-1), VEGFR-2 (Flk/KDR), and VEGFR-3 (Flt-4) expressed on the cell surface. The structure of VEGFRs, RTKs, is composed of a ligand-binding extracellular domain, a transmembrane domain, and an intracellular domain containing tyrosine kinase domain [56]. VEGF-A, the most widely studied ligand, is known to bind to both VEGFR-1 and VEGFR-2 and plays a role in angiogenesis and vascular permeability [57]. VEGFR-1 binds to VEGF-A with stronger affinity than VEGFR-2 does, but potency of tyrosine phosphorylation in response to VEGF-A is weaker than VEGFR-2 [58]. Signaling through VEGF-B is mediated by binding to VEGFR-1 and neuropilin receptors-1 (NRP-1) [59]. VEGF-C and VEGF-D bind to VEGFR-3 and are involved in lymphangiogenesis [60].

VEGFs secreted by tumor and stroma cells interact with VEGFRs mainly expressed on tumor cells. Interactions of VEGFs with their receptors stimulate angiogenesis, a process that includes proliferation and migration of endothelial cells, and remodeling of the extracellular matrix. It has been also known that VEGF triggers an epithelial-mesenchymal transition phenotype and resultant promotion of tumor invasion and survival [61].

### 3.1. Bevacizumab

Bevacizumab (Avastin®, Genentech Inc.) is a humanized monoclonal antibody directed against VEGF-A and thereby prevents VEGF-A from binding to VEGFR.

Several randomized phase II studies reported that first-line FU/leucovorin (LV) combined with bevacizumab improved treatment outcomes in patients with metastatic CRC compared with 5-FU/LV [62, 63]. A combined analysis of raw data from those studies reported improved survival in patients treated with bevacizumab plus FU/LV regimen (17.9 versus 14.6 months; HR, 0.74; *p* = 0.008) comparing with those who received FU/LV or IFL (irinotecan/fluorouracil/leucovorin) without bevacizumab [5]. In a pivotal phase III trial, metastatic CRC patients with no prior therapy were randomly assigned to receive IFL plus bevacizumab or IFL plus placebo. The primary end point was OS and a longer median duration of survival was observed in those who received IFL plus bevacizumab (20.3 versus 15.6 months; HR, 0.66; *p* < 0.001). Significantly improved median duration of PFS (10.6 versus 6.2 months; HR, 0.54; *p* < 0.001) as well as response rate (44.8% versus 34.8%, *p* = 0.004) in the bevacizumab group comparing to the placebo group was also demonstrated [64]. Efficacy of bevacizumab in combination with oxaliplatin-based chemotherapy was also examined in a large, head-to-head, randomized, double-blind, placebo-controlled, phase III study (NO 16966). Capecitabine/oxaliplatin (CapeOx) plus bevacizumab or placebo was compared with FOLFOX-4 combined with bevacizumab or placebo in 1401 patients with metastatic CRC. The addition of bevacizumab to the oxaliplatin-based chemotherapy was significantly related to the improvement of PFS (9.4 versus 8.0 months; HR, 0.83; 97.5% CI, 0.72–0.95; *p* = 0.0023) comparing with that regimen without bevacizumab. However, no difference in response rates and OS (HR, 0.89; 97.5% CI, 0.76–1.03; *p* = 0.077) was observed in this study [65]. A cohort study (ETNA) which analyzed effectiveness of bevacizumab in combination with irinotecan-based therapy as first-line treatment reported median OS of 25.3 months (95% CI, 23.3–27) [66]. Administration of FOLFIRI and bevacizumab in patients with advanced CRC as first-line treatment has also been studied. A recently reported systematic review with a pooled analysis including 3502 patients from 29 prospective and retrospective studies showed a response rate of 51.4%, a median PFS of 10.8 months (95% CI, 8.9–12.8), and a median OS of 23.7 months (95% CI, 18.1–31.6) [67]. A meta-analysis performed with 3060 patients from 6 randomized clinical trials to access the efficacy of bevacizumab used as first-line treatment in patients with metastatic CRC reported benefit of use of bevacizumab by showing results of PFS (HR, 0.72; 95% CI, 0.66–0.78; *p* < 0.00001) and OS (HR, 0.84; 95% CI, 0.77–0.91; *p* < 0.00001). Subgroup analysis, however, showed the limited benefit of irinotecan-based chemotherapy [68]. On the other hand, Passardi et al. reported results of the phase III randomized open-label clinical trial in which patients with metastatic CRC were randomized to receive first-line chemotherapy with FOLFIRI or FOLFOX4 plus bevacizumab or chemotherapy only. No benefit of the addition of bevacizumab was proven by showing results of OS (HR, 1.13; 95% CI, 0.89–1.43; *p* = 0.317) and PFS (HR, 0.86; 95% CI, 0.70–1.07; *p* = 0.182) [69] (Table 3).

Efficacy of bevacizumab in second-line treatment was analyzed in several studies (Table 4). A prospective observational cohort study (ARIES) analyzed 1550 metastatic CRC patients who received bevacizumab in combination with chemotherapy as first-line treatment and 482 patients treated with bevacizumab in second-line therapy. The median OS was 23.2 months (95% CI, 21.2–24.8) for the first-line therapy population and 17.8 months (95% CI, 16.5–20.7) for the second-line population [73]. In the phase III randomized TML (ML 18147) trial, benefit of maintenance of bevacizumab with a combination of different chemotherapy in second-line treatment after progression on bevacizumab containing first-line chemotherapy was examined. Patients with metastatic CRC were randomly assigned to receive second-line chemotherapy with or without bevacizumab. Statistically significant improvement of OS was observed in bevacizumab maintenance population (11.2 versus 9.8 months; HR, 0.81; 95% CI, 0.69–0.94; *p* = 0.0062) [70]. Another phase III randomized BEBYP trial also reported benefit of continuing bevacizumab in second-line treatment with alternative chemotherapy regimen after progression on chemotherapy containing bevacizumab by showing improved PFS (6.7 versus 5.2 months; HR, 0.66; 95% CI, 0.49–0.90; *p* = 0.0072) of the bevacizumab maintenance arm [71]. In the randomized phase III ECOG E3200 study, patients who progressed to a non-bevacizumab-containing first-line chemotherapy received FOLFOX with or without bevacizumab as second-line therapy. Improved survival was reported in patients receiving FOLFOX plus bevacizumab comparing with FOLFOX population (median OS 12.9 versus 10.8 months; *p* = 0.0011) [72]. Further studies for the mechanism of response with continuation treatment of bevacizumab in bevacizumab-failed patients should be investigated.

### 3.2. Ziv-Aflibercept

Ziv-aflibercept is a humanized recombinant fusion protein with the VEGF binding portion of human VEGFRs 1 and 2 joining the Fc portion of human IgG1. These molecules bind to VEGF-A, VEGF-B, and PLGF and subsequently result in prevention of interaction between VEGFs and their receptors, which leads to inhibition of angiogenesis.

Several preclinical studies were performed to investigate the role of aflibercept in inhibiting angiogenesis. An* in vitro* study has reported inhibition of VEGFR-2 mediated phosphorylation by aflibercept resulting in blockage of endothelial cells proliferation and angiogenesis [74]. The role of aflibercept in inhibition of tumor growth and angiogenesis and reduction of tumor vessel density in xenograft models of various tumors has also been reported in several studies [75, 76].

The double-blinded, randomized, phase III VELOUR trial assigned 1226 patients with metastatic CRC progressed to oxaliplatin-containing chemotherapy to FOLFIRI plus ziv-aflibercept or FOLFIRI plus placebo in second-line treatment. Improvement of OS was shown in FOLFIRI plus ziv-aflibercept population (13.5 versus 12.1 months; HR, 0.82; 95% CI, 0.71–0.94; *p* = 0.003) [77].

### 3.3. Ramucirumab

Ramucirumab is a human monoclonal antibody targeting the extracellular domain of VEGFR2 and interfere with VEGF signaling. Results of a phase II trial which analyzed efficacy of ramucirumab plus modified FOLFOX 6 regimen in patients with metastatic CRC showed enhanced efficacy of modified FOLFOX6 by addition of ramucirumab in first-line treatment [78]. The multicenter, randomized, double-blind, phase 3 RAISE trial was performed with metastatic CRC patients who progressed to chemotherapy comprising bevacizumab, oxaliplatin, and fluoropyrimidine by randomization to receive ramucirumab plus FOLFIRI or placebo plus FOLFIRI. Significantly improved median OS in patients receiving ramucirumab plus FOLFIRI (13.3 versus 11.7 months; HR, 0.84; 95% CI, 0.73–0.98; *p* = 0.02) was observed, meeting the primary endpoint [79]. The anti-VEGF antibodies have a stringent role in treatment of patients with metastatic CRC.

## 4. What Is Target for First Place? EGFR versus VEGF

Three representative trials were performed to compare efficacy of cetuximab or panitumumab with that of bevacizumab in first-line treatment. The randomized multicenter phase II PEAK trial compared efficacy of FOLFOX plus panitumumab with FOLFOX plus bevacizumab in patients harboring wild-type* KRAS* exon 2. PFS was revealed to be superior in the panitumumab plus FOLFOX population in the subset of 170 patients with wild-type* KRAS/NRAS* (13 versus 9.5 months; HR, 0.65; 95% CI, 0.44–0.96; *p* = 0.03) [80]. The open-label, randomized, multicenter FIRE-3 trial assigned 592 patients with* KRAS* exon 2 wild-type metastatic CRC to FOLFIRI plus cetuximab or FOLFIRI plus bevacizumab in first-line treatment. No significant difference in ORR, the primary endpoint of this study, was observed (62.0% versus 58.0%; *p* = 0.18), although OS was reported to be significantly increased in the cetuximab group (28.7 versus 25.0 months; HR, 0.77; 95% CI, 0.62–0.96; *p* = 0.017) [7]. The phase III CALGB/SWOG 80405 trial addressed the optimal antibody combination with chemotherapy. Patients with wild-type* KRAS* exon 2 receiving FOLFOX or FOLFIRI were randomly assigned to have cetuximab or bevacizumab. No significantly different OS was reported between cetuximab and bevacizumab population (HR, 0.92; 95% CI, 0.78–1.09, *p* = 0.34). Until now, there is no winner at first-line chemotherapy for metastatic colon cancer. Therefore, choice of chemotherapy should be based on side effects and tolerability.

## 5. Possible Chemotherapies according to Clinical Subtypes

Because the goal of treatment is different according to clinical subtypes in metastatic CRC, differentiated choice of appropriate chemotherapeutic regimens should be taken into consideration at the time of establishment of treatment plan.

Both cetuximab and panitumumab plus chemotherapies such as FOLFOX or FOLFIRI are the feasible regimens as the induction therapy for conversion to resectable status in patients with potentially resectable metastatic CRC harboring wild-type* RAS* [16–18]. In addition, efficacy of the addition of bevacizumab to FOLFOXIRI (infusional 5-FU, LV, oxaliplatin, and irinotecan) reported in two randomized clinical trials is also quite encouraging. In Gruppo Oncologico Nord Ovest's (GONO) phase III TRIBE trial, the ORR was 65% in the FOLFOXIRI plus bevacizumab group and 53% in the FOLFIRI plus bevacizumab group (*p* = 0.006) [81]. The randomized phase II OLIVIA trial reported increased* R*0 resection rate in FOLFOXIRI plus bevacizumab group comparing with mFOLFOX6 plus bevacizumab group (49% versus 23%; 95% CI, 4–48%) [82]. Despite the proven efficacy, FOFOXIRI is reported to be related to higher frequencies of grade 3 or 4 toxicities in terms of neutropenia, diarrhea, stomatitis, and neurotoxicity in those two studies. Considering those results collectively, anti-EGFR antibodies combined with chemotherapy could be adopted as the induction chemotherapy. FOLFOXIRI plus bevacizumab could also be an option in consideration of its efficacy, but significant adverse effects should be taken into account so that limited use of the regimen in selected patients would be reasonable.

Patients who need palliative chemotherapies with good performance status are required to be treated with active chemotherapeutic regimens including targeted agents given the aggressive biological feature. Three head-to-head trials showed equivalent efficacy between treatments with anti-EGFR antibodies and bevacizumab in terms of their primary endpoint [7, 32, 80]. Considering the proven efficacy of bevacizumab in early phase of continuum of care and effectiveness of the anti-EGFR monoclonal antibody in the later line of treatment in patients with metastatic CRC, use of bevacizumab in combination with chemotherapy as the first-line therapy could be an option [19, 20, 70, 71]. Although Passardi et al. reported no benefit of bevacizumab as the front-line treatment in combination with FOLFIRI or FOLFOX4 in a phase III randomized trial, there is a limitation that only a small number of patients were analyzed in this study [69]. Currently, either one of those targeted agents, anti-EGFR monoclonal antibodies or bevacizumab, is regarded to be a reasonable option to use as the initial line of treatment in combination with FOLFOX or FOLFIRI. Because an appropriate sequence of use of targeted agents has not been determined, an ongoing phase III clinical study is trying to access the optimal use and the best sequencing of the targeted therapies. The randomized, open-label STRATEGIC-1 phase III trial comparing two treatment strategies, first-line FOLFIRI-cetuximab followed by second-line oxaliplatin-based chemotherapy with bevacizumab (Arm A) versus oxaliplatin-based chemotherapy plus bevacizumab as first-line followed by irinotecan-based second-line chemotherapy plus bevacizumab and third line anti-EGFR monoclonal antibody with or without irinotecan (Arm B), is currently being undergone [83]. On the other hand, FOFOXIRI combined with bevacizumab is also another option as the first-line treatment in selected patients for the significant adverse effects.

For patients with poor performance status with symptoms of tumor burden, given that goal of treatment is prolongation of life with palliation of symptoms by reducing tumor burden, careful selection of chemotherapeutic agents is required based on benefit and disadvantages. Anti-EGFR antibodies or bevacizumab in combination with chemotherapy is also a choice for patients in this group.

## 6. Resistance Mechanisms to Anti-EGFR Therapy

Encouraged by the improved outcomes of treatment with anti-EGFR monoclonal antibodies, addition of the EGFR-directed monoclonal antibodies to chemotherapy has been the standard therapy in a subset of patients with* KRAS/NRAS* wild-type metastatic CRC. However, patients responsive to the targeted therapy have been known to ultimately acquire resistance. One of the mechanisms of resistance to anti-EGFR therapies is acquisition of mutations in* EGFR*.

A point mutation (S492R) at the extracellular domain of EGFR found in a cetuximab-resistant CRC cell line was reported to prevent the antibody from binding to EGFR in a study. The study reported that 2 of 10 subjects who progressed to cetuximab treatment were revealed to harbor the S492R mutation. Despite the proven resistance to the cetuximab, the patient with S492R mutation was shown to be responsive to panitumumab [84].

Another reported mechanism for resistance to anti-EGRF antibodies is amplification of genes that encode RTKs. Both* de novo* and acquired amplification of* ERBB2* or* MET* gene were reported in patients with metastatic CRC who showed resistance to the anti-EGFR therapy [85, 86].

Mutations in* RAS* genes have also been suggested as a mechanism for resistance to cetuximab or panitumumab. Circulating cell-free tumor DNA from plasma of 24 patients with CRC at recurrence and before treatment with anti-EGFR antibodies was analyzed for genetic alterations in* RAS* genes. In total, 70 new mutations after the EGFR blockade were found. Half of the newly detected mutations were in codon 12 or 13 of KRAS; mutations in BRAF (V600E) were also observed in two patients; mutations in EGFR kinase domain were detected in two patients [87].

## 7. Multiple Receptors Kinases Inhibitor

*Regorafenib*. Regorafenib is a multikinase inhibitor that blocks the activity of protein kinases of several receptors (VEGFR1, VEGFR2, VEGFR3, TIE2, KIT, RET, RAF1, BRAF, PDGFR, and FGFR) involved in various signaling pathways regulating angiogenesis, tumor growth, and tumor microenvironment [88]. In the international, multicenter, randomized, placebo-controlled phase III CORRECT trial, patients with metastatic CRC who progressed to the standard therapy were assigned to receive the best supportive care plus regorafenib or placebo. This trial proved the benefit of regorafenib by showing prolonged OS in patients who received regorafenib (6.4 versus 5 months; HR, 0.77; 95% CI, 0.64–0.94; *p* = 0.005) [89]. Another study which evaluated efficacy of regorafenib in Asian patients also reported benefit of this multikinase inhibitor. A randomized, double-blind, placebo-controlled, phase III CONCUR trial randomized Asian patients with progressive metastatic CRC who had received at least two previous treatment lines to have regorafenib plus best supportive care or placebo plus best supportive care. No prior use of target agents before enrollment was mandatory, and around 40% of enrolled patients were not exposed to targeted agents. Significant survival advantage was shown in regorafenib group meeting the primary endpoint (8.8 versus 6.3 months; HR, 0.55; 95% CI, 0.40–0.77; one-sided *p* = 0.00016) [90]. This study showed that exposure to targeted agents was not prerequirement to regorafenib treatment.

## 8. New Targeted Therapy

We summarized the mechanism of action of biologic agents currently used in treatment of CRC and historical studies which evaluated the efficacy of those agents. Unfortunately, despite the improvement of treatments outcomes in patients with metastatic CRC by application of biologic agents to clinical practice, their prognosis still remains dismal. Efforts to overcome the limited efficacy of current therapy are ongoing, and studies with new biologic agents are in progress.

### 8.1. EGFR Tyrosine Kinase Inhibitor

EGFR tyrosine kinase inhibitors (TKIs) (erlotinib or gefitinib) are directed to intracellular tyrosine kinase domain of the receptor. Unlike lung cancer, treatment with TKI in combination of chemotherapy has been reported to be ineffective in CRC. A randomized phase II trial which examined efficacy of FOLFIRI with or without gefitinib reported disappointing results with no improvement in ORR or OS in gefitinib population [91]. However, the randomized phase III DREAM trial showed that the addition of erlotinib to bevacizumab maintenance therapy after bevacizumab-based induction therapy with FOLFOX or XELOX or FOLFIRI resulted in significant improvement in PFS (4.6 versus 5.8 months; HR, 0.73; 95% CI, 0.59–0.91; *p* = 0.005) [92].

Another clinical trial to see efficacy of dual EGFR blockade in the presence of erlotinib and panitumumab with or without chemotherapy for advanced CRC is currently being performed with patients harboring wild-type* KRAS* gene (NCT00940316).

### 8.2. BRAF Inhibitors: Vemurafenib

BRAF^V600E^ mutation, occupying 10% of CRC, is known to be blocked by vemurafenib. However, despite the proven efficacy in treatment of advanced melanoma, the role of vemurafenib in CRC remains to be elusive. A preclinical study found that the antitumor activity of vemurafenib in a V600E CRC model was potentiated by combined use of EGFR inhibitors [93]. Based on the finding, several clinical studies have been performed. The combination of vemurafenib and panitumumab has been examined for its efficacy in patients with BRAF^V600E^ mutated metastatic CRC, and tumor regression of >15% by response evaluation criteria in solid tumors (RECIST) measurement was observed in 8 of 15 patients [94] (NCT01791309). A phase II trial to see efficacy of irinotecan plus cetuximab with or without vemurafenib is currently comparing those two groups in patients with* BRAF* mutation who progressed to one or two prior lines of chemotherapies (NCT02164916).

### 8.3. MEK Inhibitor: Selumetinib

A multicenter open-label phase I/II trial evaluated efficacy of the combination therapy of irinotecan plus selumetinib, a small molecule kinase inhibitor targeting MEK kinase, in patients with metastatic CRC harboring* KRAS* mutation progressed on the oxaliplatin-based regimen with bevacizumab. The primary endpoint was RECIST response rate. Three of 31 (9.7%) patients had partial response, and 16 (51.6%) patients showed stable disease. These results were concluded to be promising comparing with historical controls [95].

### 8.4. Antiangiogenic Agent: Famitinib

Famitinib is a small molecule inhibitor that blocks multiple receptors tyrosine kinases including VEGFR2, VEGFR3, PDGFR, c-KIT, FLT3, and RET. Recently reported results from a multicenter, randomized, double-blind, phase II study which analyzed efficacy of famitinib demonstrated benefit of this agent. Patients with metastatic CRC who failed second- or later-line treatments were randomized to receive famitinib or placebo. Improved PFS was shown in patients assigned to receive famitinib (2.8 versus 1.5 months; HR, 0.58; *p* = 0.0034), meeting the primary endpoint [96].

### 8.5. Anti-Programmed Death 1 Immune Checkpoint Inhibitor: Pembrolizumab

Pembrolizumab is an anti-programmed death 1 (PD-1) immune checkpoint inhibitor that blocks the PD-1 pathway, a negative feedback system repressing Th1 cytotoxic immune responses. A phase II trial to evaluate the efficacy of pembrolizumab in patients with progressive metastatic carcinoma refractory to previous therapies with or without mismatch-repair (MMR) deficiency reported benefit of this agent in patients with MMR deficiency. In patients with CRC, the immune-related ORR and immune-related PFS rate were 40% and 78%, respectively, for MMR-deficient CRC and 0% and 11% for MMR-proficient CRC [97].

## 9. Prognostic Models in the Era of Targeted Therapies

The Köhne and GERCOR risk classifications are two representative prognostic models which subdivide patients with CRC into three risk groups. The Köhne model was established with metastatic CRC patients treated with 5-FU-based chemotherapy. The risk group was classified according to patient-, biology-, or tumor-related factors. Performance status (PS), white blood cell count, alkaline phosphatase (ALP), and number of metastatic sites or liver invasion are factors taken into account in classification of risk groups [98]. Afterwards the GERCOR prognostic model was developed for patients with metastatic CRC treated with oxaliplatin- or irinotecan-based first-line chemotherapy. Based on two clinical parameters, serum lactate dehydrogenase (LDH) level, and PS, a more simplified prognostic model was established [99]. The relevancy of the Köhne prognostic model to patients treated with targeted biologic agents was addressed in several studies. A* post hoc* analysis of patients involved in the phase III trial comparing IFL plus bevacizumab to placebo [64] and in the combined analysis of 5-FU/LV plus bevacizumab or placebo [5] reported that the Köhne model is also applicable to patients treated with bevacizumab plus FU-based chemotherapy by showing improved OS and PFS across the Köhne risk classification [100]. In subgroup analyses, however, it revealed that median OS in the intermediate-risk group in patients receiving 5-FU/LV with or without bevacizumab was not significantly different. In addition, lower median PFS of intermediate-risk group compared to that of high-risk group in patients receiving 5-FU/LV plus bevacizumab was observed. Another study exploring validity of the Köhne classification in patients with metastatic CRC in whom approximately 30% received targeted biotherapies reported the questioning relevance of the model in the era of biotherapies [101]. For the limited reports on the relevance of those prognostic classifications and biologic benefit of targeted agents, further study is necessary to define the role of those models in the era of targeted therapies.

Besides those prognosis classifications, a recently reported molecular classification addressed its relevance with clinical response to cetuximab. Sadanandam et al. subdivided CRC into six subtypes, stem-like, inflammatory, cetuximab-sensitive transit-amplifying (CS-TA), cetuximab-resistant transit-amplifying (CR-TA), goblet-like, and enterocyte subtype, based on the gene expression profiles and differential response to cetuximab. The authors explored responsiveness of cetuximab on the segregation to see biological benefit of the agent. CS-TA subtype was shown to be sensitive to the agent in both* in vitro* and* in vivo* xenograft models [102]. Collectively, from the results, CS-TA subtype might be successfully treated with cetuximab in metastatic CRC and could be a guide in application of cetuximab in addition to* RAS* mutations, but these outcomes should be demonstrated further by retrospective and prospective studies.

## 10. Conclusion

Although enormous progress has been made in treatment of metastatic CRC, the prognosis still remains poor. In this review, we summarized representative studies which have brought change of stream of therapy in patients with CRC. The watershed of improvement of treatment outcomes has been the introduction of biologic agents such as anti-EGFR monoclonal antibodies or antiangiogenic agents. Application of biologic agents to patients extended median survival up to over 2 years, and the combination chemotherapy with conventional chemotherapeutic and targeted agents has been established as the standard therapy. However, resistance to the targeted agents has emerged as a new issue to overcome in recent years. The acquired mutations have been proposed as one of reasons for the refractoriness of colorectal tumors to biologic agents. Therefore, further clinical trials for targeting these mutations should be considered. Furthermore, several clinical trials to examine efficacy of the genomic sequencing guided individualized therapy are being underwent currently. A continuous effort will be devoted to improve outcomes of treatment in CRC by clarifying mechanisms of oncogenesis and developing new chemicals, and attention should be paid to not only results of preclinical studies but also outcomes of ongoing clinical studies.

## Figures and Tables

**Table 1:** Clinical trials with anti-EGFR monoclonal antibodies in postprogression treatment.

Study	No. of patients	Design	Treatment	Primary end point	Results	*p*
BOND [19]	329	Phase 3, open-label, RCT	C-mab versus C-mab + irinotecan	ORR	10.8% versus 22.9%	0.007

CO17 [20]	572	Phase 3, RCT	BSC versus C-mab	OS	HR, 0.77; 95% CI, 0.64–0.92	0.005

EPIC [21]	1298	Phase 3, open-label, RCT	Irinotecan versus C-mab + irinotecan	OS	HR, 0.975; 95% CI, 0.85–1.11	0.71

Van Cutsem et al. [22]	463	Phase III, open-label, RCT	BSC versus P-mab + BSC	PFS	HR, 0.54; 95% CI, 0.44–0.66	<0.0001

Peeters et al. [23]	1186	Phase III, open-label, RCT	FOLFIRI versus P-mab + FOLFIRI	PFS	HR, 0.73; 95% CI, 0.59–0.9	0.004
				OS	HR, 0.85; 95% CI, 0.7–1.04	0.12

PICCOLO [26]	460	Phase III, open-label, RCT	Irinotecan versus P-mab + irinotecan	OS	HR, 1.01; 95% CI, 0.83–1.23	0.91

EGFR: epidermal growth factor receptor; No.: number; RCT: randomized controlled trial; pt: patient; C-mab: cetuximab; ORR: objective response rate; BSC: best supportive care; OS: overall survival; HR: hazard ratio; P-mab: panitumumab; PFS: progression-free survival.

**Table 2:** Clinical trials with anti-EGFR monoclonal antibodies in first-line treatment.

Study	No. of patients	Design	Treatment	Primary end point	Results	*p*
CRYSTAL [27]	1198	Phase 3, open-label, RCT	FOLFIRI versus C-mab + FOLFIRI	PFS	HR, 0.85; 95% CI, 0.72–0.99	0.048

OPUS [28]	337	Phase 2, open-label, RCT	FOLFOX4 versus C-mab + FOLFOX4	ORR	37% versus 61%^*∗*^	0.011

MRC COIN [29]	1630	Phase III, open-label, RCT	FOLFOX versus C-mab + FOLFOX	OS	HR, 1.04; 95% CI, 0.87–1.23^*∗*^	0.67
			CapeOx CapeOx			

NORDIC VII [30]	571	Phase III, open-label, RCT	Nordic FLOX versus C-mab + FLOX versus C-mab + intermittent FLOX	PFS	HR, 0.89; 95% CI, 0.72–1.11	0.31

PRIME [31]	1183	Phase III, open-label, RCT	FOLFOX4 versus P-mab + FOLFOX4	PFS	HR, 0.8; 95% CI, 0.66–0.97^*∗*^	0.02

^*∗*^Results were analyzed with tumors harboring wild-type exon 2 *KRAS*

EGFR: epidermal growth factor receptor; No., number; RCT: randomized controlled trial; C-mab: cetuximab; PFS: progression-free survival; HR: hazard ratio; ORR: objective response rate; OS: overall survival; CapeOx: capecitabine plus oxaliplatin; P-mab: panitumumab.

**Table 3:** Clinical trials with bevacizumab in first-line treatment.

Study	No. of patients	Design	Treatment	Primary end point	Results	*p*
Kabbinavar et al. [62]	104	Phase 2, randomized	FU/LV versus	TTP	5.2 versus 7.4 months	0.013
			Low dose bevacizumab + FU/LV High dose bevacizumab + FU/LV	Best response rate	17% versus 32%	0.086

Kabbinavar et al. [63]	209	Phase 2, randomized	FU/LV + placebo versus FU/LV + bevacizumab	OS	12.9 versus 16.6 months; HR, 0.79	0.16

Hurwitz et al. [64]	813	Phase 3, double-blind, RCT	IFL + placebo versus IFL + bevacizumab	OS	15.6 versus 20.3 months; HR, 0.66	<0.001

NO 16966 [65]	1401	Phase 3, double-blind, RCT	CapeOx + placebo or CapOx + bevacizumab versus FOLFOX + placebo or FOLFOX + bevacizumab	PFS	HR, 0.83; 95% CI, 0.72–0.95	0.0023

Passardi et al. [69]	376	Phase 3, randomized	FOLFIRI or FOLFOX + bevacizumab versus FOLFIRI or FOLFOX	PFS	HR, 0.86; 95% CI, 0.70–1.07	0.182

No.: number; TTP: time to progression; RCT: randomized controlled trial; OS: overall survival; HR: hazard ratio; PFS: progression-free survival; CI: confidence interval.

**Table 4:** Clinical trials with bevacizumab as second-line treatment.

Study	No. of patients	Design	Treatment	Primary end point	Results	*p*
TML [70]	820	Phase 3, open-label, RCT	CTx versus bevacizumab + CTx	OS	HR, 0.81; 95% CI, 0.69–0.94	0.0062

BEBYP [71]	185	Phase III, RCT	FOLFIRI or mFOLFOX6 versus FOLFIRI or mFOLFOX6 + bevacizumab	PFS	HR, 0.66; 95% CI, 0.49–0.90	0.0072

ECOG E3200 [72]	829	Phase III, open-label, RCT	FOLFOX4 + bevacizumab versus FOLFOX4 versus bevacizumab	OS	HR, 0.75	0.0011

No.: number; PFS: progression-free survival; CI: confidence interval; RCT: randomized controlled trial; HR: hazard ratio; OS: overall survival; CTx: chemotherapy.

## References

[1] Siegel R., Naishadham D., Jemal A. (2013). Cancer statistics, 2013. *CA Cancer Journal for Clinicians*.

[2] Muratore A., Zorzi D., Bouzari H. (2007). Asymptomatic colorectal cancer with un-resectable liver metastases: immediate colorectal resection or up-front systemic chemotherapy?. *Annals of Surgical Oncology*.

[3] Kemeny N., Fata F. (1999). Arterial, portal, or systemic chemotherapy for patients with hepatic metastasis of colorectal carcinoma. *Journal of Hepato-Biliary-Pancreatic Surgery*.

[4] Piedbois P., Rougier P., Buyse M. (1998). Efficacy of intravenous continuous infusion of fluorouracil compared with bolus administration in advanced colorectal cancer. *Journal of Clinical Oncology*.

[5] Kabbinavar F. F., Hambleton J., Mass R. D., Hurwitz H. I., Bergsland E., Sarkar S. (2005). Combined analysis of efficacy: the addition of bevacizumab to fluorouracil/leucovorin improves survival for patients with metastatic colorectal cancer. *Journal of Clinical Oncology*.

[6] Van Cutsem E., Köhne C.-H., Láng I. (2011). Cetuximab plus irinotecan, fluorouracil, and leucovorin as first-line treatment for metastatic colorectal cancer: updated analysis of overall survival according to tumor KRAS and BRAF mutation status. *Journal of Clinical Oncology*.

[7] Heinemann V., von Weikersthal L. F., Decker T. (2014). Folfiri plus cetuximab versus folfiri plus bevacizumab as first-line treatment for patients with metastatic colorectal cancer (fire-3): a randomised, open-label, phase 3 trial. *The Lancet Oncology*.

[8] Casalini P., Iorio M. V., Galmozzi E., Ménard S. (2004). Role of HER receptors family in development and differentiation. *Journal of Cellular Physiology*.

[9] Prenzel N., Fischer O. M., Streit S., Hart S., Ullrich A. (2001). The epidermal growth factor receptor family as a central element for cellular signal transduction and diversification. *Endocrine-Related Cancer*.

[10] Rodriguez-Viciana P., Warne P. H., Dhand R. (1994). Phosphatidylinositol-3-OH kinase as a direct target of Ras. *Nature*.

[11] Datta S. R., Brunet A., Greenberg M. E. (1999). Cellular survival: a play in three akts. *Genes & Development*.

[12] Kelley G. G., Reks S. E., Ondrako J. M., Smrcka A. V. (2001). Phospholipase C*ε*: a novel Ras effector. *The EMBO Journal*.

[13] Downward J. (2003). Targeting RAS signalling pathways in cancer therapy. *Nature Reviews Cancer*.

[14] Recondo G., Diaz-Canton E., de la Vega M., Greco M., Recondo G., Valsecchi M. E. (2014). Advances and new perspectives in the treatment of metastatic colon cancer. *World Journal of Gastrointestinal Oncology*.

[15] Prewett M. C., Hooper A. T., Bassi R., Ellis L. M., Waksal H. W., Hicklin D. J. (2002). Enhanced antitumor activity of anti-epidermal growth factor receptor monoclonal antibody IMC-C225 in combination with irinotecan (CPT-11) against human colorectal tumor xenografts. *Clinical Cancer Research*.

[16] Folprecht G., Gruenberger T., Bechstein W. O. (2010). Tumour response and secondary resectability of colorectal liver metastases following neoadjuvant chemotherapy with cetuximab: the CELIM randomised phase 2 trial. *The Lancet Oncology*.

[17] Ye L.-C., Liu T.-S., Ren L. (2013). Randomized controlled trial of cetuximab plus chemotherapy for patients with KRAS wild-type unresectable colorectal liver-limited metastases. *Journal of Clinical Oncology*.

[18] Petrelli F., Barni S., Anti-EGFR Agents for Liver Metastases (2012). Resectability and outcome with anti-EGFR agents in patients with KRAS wild-type colorectal liver-limited metastases: a meta-analysis. *International Journal of Colorectal Disease*.

[19] Cunningham D., Humblet Y., Siena S. (2004). Cetuximab monotherapy and cetuximab plus irinotecan in irinotecan-refractory metastatic colorectal cancer. *The New England Journal of Medicine*.

[20] Jonker D. J., O'Callaghan C. J., Karapetis C. S. (2007). Cetuximab for the treatment of colorectal cancer. *The New England Journal of Medicine*.

[21] Sobrero A. F., Maurel J., Fehrenbacher L. (2008). EPIC: phase III trial of cetuximab plus irinotecan after fluoropyrimidine and oxaliplatin failure in patients with metastatic colorectal cancer. *Journal of Clinical Oncology*.

[22] Van Cutsem E., Peeters M., Siena S. (2007). Open-label phase III trial of panitumumab plus best supportive care compared with best supportive care alone in patients with chemotherapy-refractory metastatic colorectal cancer. *Journal of Clinical Oncology*.

[23] Peeters M., Price T. J., Cervantes A. (2010). Randomized phase III study of panitumumab with fluorouracil, leucovorin, and irinotecan (FOLFIRI) compared with FOLFIRI alone as second-line treatment in patients with metastatic colorectal cancer. *Journal of Clinical Oncology*.

[24] Amado R. G., Wolf M., Peeters M. (2008). Wild-type kras is required for panitumumab efficacy in patients with metastatic colorectal cancer. *Journal of Clinical Oncology*.

[25] Peeters M., Price T. J., Cervantes A. (2014). Final results from a randomized phase 3 study of FOLFIRI ± panitumumab for second-line treatment of metastatic colorectal cancer. *Annals of Oncology*.

[26] Seymour M. T., Brown S. R., Middleton G. (2013). Panitumumab and irinotecan versus irinotecan alone for patients with KRAS wild-type, fluorouracil-resistant advanced colorectal cancer (PICCOLO): a prospectively stratified randomised trial. *The Lancet Oncology*.

[27] Van Cutsem E., Köhne C.-H., Hitre E. (2009). Cetuximab and chemotherapy as initial treatment for metastatic colorectal cancer. *The New England Journal of Medicine*.

[28] Bokemeyer C., Bondarenko I., Makhson A. (2009). Fluorouracil, leucovorin, and oxaliplatin with and without cetuximab in the first-line treatment of metastatic colorectal cancer. *Journal of Clinical Oncology*.

[29] Maughan T. S., Adams R. A., Smith C. G. (2011). Addition of cetuximab to oxaliplatin-based first-line combination chemotherapy for treatment of advanced colorectal cancer: results of the randomised phase 3 MRC COIN trial. *The Lancet*.

[30] Tveit K. M., Guren T., Glimelius B. (2012). Phase iii trial of cetuximab with continuous or intermittent fluorouracil, leucovorin, and oxaliplatin (Nordic FLOX) versus FLOX alone in first-line treatment of metastatic colorectal cancer: the NORDIC-VII study. *Journal of Clinical Oncology*.

[31] Douillard J.-Y., Siena S., Cassidy J. (2010). Randomized, phase III trial of panitumumab with infusional fluorouracil, leucovorin, and oxaliplatin (FOLFOX4) versus FOLFOX4 alone as first-line treatment in patients with previously untreated metastatic colorectal cancer: the prime study. *Journal of Clinical Oncology*.

[32] Venook A. P., Niedzwiecki D., Lenz H. J. (2014). CALGB/SWOG 80405: phase III trial of irinotecan/5-FU/leucovorin (FOLFIRI) or oxaliplatin/5-FU/leucovorin (mFOLFOX6) with bevacizumab (BV) or cetuximab (CET) for patients (pts) with KRAS wild-type (wt) untreated metastatic adenocarcinoma of the colon or rectum (MCRC). *Journal of Clinical Oncology*.

[33] Douillard J.-Y., Oliner K. S., Siena S. (2013). Panitumumab-FOLFOX4 treatment and RAS mutations in colorectal cancer. *The New England Journal of Medicine*.

[34] Antonacopoulou A. G., Tsamandas A. C., Petsas T. (2008). Egfr, her-2 and cox-2 levels in colorectal cancer. *Histopathology*.

[35] McKay J. A., Murray L. J., Curran S. (2002). Evaluation of the epidermal growth factor receptor (EGFR) in colorectal tumours and lymph node metastases. *European Journal of Cancer*.

[36] Spano J.-P., Lagorce C., Atlan D. (2005). Impact of EGFR expression on colorectal cancer patient prognosis and survival. *Annals of Oncology*.

[37] Yen L.-C., Uen Y.-H., Wu D.-C. (2010). Activating kras mutations and overexpression of epidermal growth factor receptor as independent predictors in metastatic colorectal cancer patients treated with cetuximab. *Annals of Surgery*.

[38] Chung K. Y., Shia J., Kemeny N. E. (2005). Cetuximab shows activity in colorectal cancer patients with tumors that do not express the epidermal growth factor receptor by immunohistochemistry. *Journal of Clinical Oncology*.

[39] Hecht J. R., Mitchell E., Neubauer M. A. (2010). Lack of correlation between epidermal growth factor receptor status and response to panitumumab monotherapy in metastatic colorectal cancer. *Clinical Cancer Research*.

[40] Saltz L. B., Meropol N. J., Loehrer P. J., Needle M. N., Kopit J., Mayer R. J. (2004). Phase II trial of cetuximab in patients with refractory colorectal cancer that expresses the epidermal growth factor receptor. *Journal of Clinical Oncology*.

[41] Karapetis C. S., Khambata-Ford S., Jonker D. J. (2008). K-ras mutations and benefit from cetuximab in advanced colorectal cancer. *The New England Journal of Medicine*.

[42] Lièvre A., Bachet J.-B., Boige V. (2008). KRAS mutations as an independent prognostic factor in patients with advanced colorectal cancer treated with cetuximab. *Journal of Clinical Oncology*.

[43] Edkins S., O'Meara S., Parker A. (2006). Recurrent KRAS codon 146 mutations in human colorectal cancer. *Cancer Biology and Therapy*.

[44] Janakiraman M., Vakiani E., Zeng Z. (2010). Genomic and biological characterization of exon 4 KRAS mutations in human cancer. *Cancer Research*.

[45] Smith G., Bounds R., Wolf H., Steele R. J. C., Carey F. A., Wolf C. R. (2010). Activating K-Ras mutations outwith hotspot codons in sporadic colorectal tumours-implications for personalised cancer medicine. *British Journal of Cancer*.

[46] Vaughn C. P., Zobell S. D., Furtado L. V., Baker C. L., Samowitz W. S. (2011). Frequency of KRAS, BRAF, and NRAS mutations in colorectal cancer. *Genes Chromosomes and Cancer*.

[47] Tol J., Nagtegaal I. D., Punt C. J. A. (2009). BRAF mutation in metastatic colorectal cancer. *New England Journal of Medicine*.

[48] Price T. J., Hardingham J. E., Lee C. K. (2011). Impact of KRAS and BRAF gene mutation status on outcomes from the phase III AGITG MAX trial of capecitabine alone or in combination with bevacizumab and mitomycin in advanced colorectal cancer. *Journal of Clinical Oncology*.

[49] Clancy C., Burke J. P., Kalady M. F., Coffey J. C. (2013). BRAF mutation is associated with distinct clinicopathological characteristics in colorectal cancer: a systematic review and meta-analysis. *Colorectal Disease*.

[50] Di Nicolantonio F., Martini M., Molinari F. (2008). Wild-type BRAF is required for response to panitumumab or cetuximab in metastatic colorectal cancer. *Journal of Clinical Oncology*.

[51] Laurent-Puig P., Cayre A., Manceau G. (2009). Analysis of PTEN, BRAF, and EGFR status in determining benefit from cetuximab therapy in wild-type KRAS metastatic colon cancer. *Journal of Clinical Oncology*.

[52] Loupakis F., Ruzzo A., Cremolini C. (2009). KRAS codon 61, 146 and BRAF mutations predict resistance to cetuximab plus irinotecan in KRAS codon 12 and 13 wild-type metastatic colorectal cancer. *British Journal of Cancer*.

[53] De Roock W., Claes B., Bernasconi D. (2010). Effects of KRAS, BRAF, NRAS, and PIK3CA mutations on the efficacy of cetuximab plus chemotherapy in chemotherapy-refractory metastatic colorectal cancer: a retrospective consortium analysis. *The Lancet Oncology*.

[54] Seymour M. T., Brown S. R., Richman S. (2011). Addition of panitumumab to irinotecan: results of PICCOLO, a randomized controlled trial in advanced colorectal cancer (aCRC). *Journal of Clinical Oncology*.

[55] Neufeld G., Cohen T., Gengrinovitch S., Poltorak Z. (1999). Vascular endothelial growth factor (VEGF) and its receptors. *The FASEB Journal*.

[56] Yancopoulos G. D., Davis S., Gale N. W., Rudge J. S., Wiegand S. J., Holash J. (2000). Vascular-specific growth factors and blood vessel formation. *Nature*.

[57] Senger D. R. (2010). Vascular endothelial growth factor: much more than an angiogenesis factor. *Molecular Biology of the Cell*.

[58] Koch S., Claesson-Welsh L. (2012). Signal transduction by vascular endothelial growth factor receptors. *Cold Spring Harbor Perspectives in Medicine*.

[59] Holmqvist K., Cross M. J., Rolny C. (2004). The adaptor protein Shb binds to tyrosine 1175 in vascular endothelial growth factor (VEGF) receptor-2 and regulates VEGF-dependent cellular migration. *The Journal of Biological Chemistry*.

[60] Karkkainen M. J., Petrova T. V. (2000). Vascular endothelial growth factor receptors in the regulation of angiogenesis and lymphangiogenesis. *Oncogene*.

[61] Goel H. L., Mercurio A. M. (2013). VEGF targets the tumour cell. *Nature Reviews Cancer*.

[62] Kabbinavar F., Hurwitz H. I., Fehrenbacher L. (2003). Phase II, randomized trial comparing bevacizumab plus fluorouracil (FU)/Leucovorin (LV) with FU/LV alone in patients with metastatic colorectal cancer. *Journal of Clinical Oncology*.

[63] Kabbinavar F. F., Schulz J., McCleod M. (2005). Addition of bevacizumab to bolus fluorouracil and leucovorin in first-line metastatic colorectal cancer: results of a randomized phase II trial. *Journal of Clinical Oncology*.

[64] Hurwitz H., Fehrenbacher L., Novotny W. (2004). Bevacizumab plus irinotecan, fluorouracil, and leucovorin for metastatic colorectal cancer. *The New England Journal of Medicine*.

[65] Saltz L. B., Clarke S., Díaz-Rubio E. (2008). Bevacizumab in combination with oxaliplatin-based chemotherapy as first-line therapy in metastatic colorectal cancer: a randomized phase III study. *Journal of Clinical Oncology*.

[66] Fourrier-Réglat A., Smith D., Rouyer M. (2014). Survival outcomes of bevacizumab in first-line metastatic colorectal cancer in a real-life setting: results of the ETNA cohort. *Targeted Oncology*.

[67] Petrelli F., Borgonovo K., Cabiddu M. (2013). FOLFIRI-bevacizumab as first-line chemotherapy in 3500 patients with advanced colorectal cancer: a pooled analysis of 29 published trials. *Clinical Colorectal Cancer*.

[68] Macedo L. T., da Costa Lima A. B., Sasse A. D. (2012). Addition of bevacizumab to first-line chemotherapy in advanced colorectal cancer: a systematic review and meta-analysis, with emphasis on chemotherapy subgroups. *BMC Cancer*.

[69] Passardi A., Nanni O., Tassinari D. (2015). Effectiveness of bevacizumab added to standard chemotherapy in metastatic colorectal cancer: final results for first-line treatment from the ITACa randomized clinical trial. *Annals of Oncology*.

[70] Bennouna J., Sastre J., Arnold D. (2013). Continuation of bevacizumab after first progression in metastatic colorectal cancer (ML18147): a randomised phase 3 trial. *The Lancet Oncology*.

[71] Masi G., Loupakis F., Salvatore L. (2013). Second-line chemotherapy (ct) with or without bevacizumab (bv) in metastatic colorectal cancer (mcrc) patients (pts) who progressed to a first-line treatment containing bv: updated results of the phase iii “bebyp” trial by the gruppo oncologico nord ovest (gono). *Journal of Clinical Oncology*.

[72] Giantonio B. J., Catalano P. J., Meropol N. J. (2007). Bevacizumab in combination with oxaliplatin, fluorouracil, and leucovorin (FOLFOX4) for previously treated metastatic colorectal cancer: results from the Eastern Cooperative Oncology Group study E3200. *Journal of Clinical Oncology*.

[73] Hurwitz H. I., Bekaii-Saab T. S., Bendell J. C. (2014). Safety and effectiveness of bevacizumab treatment for metastatic colorectal cancer: final results from the avastin® registry—investigation of effectiveness and safety (ARIES) observational cohort study. *Clinical Oncology*.

[74] Byrne A. T., Ross L., Holash J. (2003). Vascular endothelial growth factor-trap decreases tumor burden, inhibits ascites, and causes dramatic vascular remodeling in an ovarian cancer model. *Clinical Cancer Research*.

[75] Verheul H. M. W., Hammers H., van Erp K. (2007). Vascular endothelial growth factor trap blocks tumor growth, metastasis formation, and vascular leakage in an orthotopic murine renal cell cancer model. *Clinical Cancer Research*.

[76] Fukasawa M., Korc M. (2004). Vascular endothelial growth factor-trap suppresses tumorigenicity of multiple pancreatic cancer cell lines. *Clinical Cancer Research*.

[77] Van Cutsem E., Tabernero J., Lakomy R. (2012). Addition of aflibercept to fluorouracil, leucovorin, and irinotecan improves survival in a phase III randomized trial in patients with metastatic colorectal cancer previously treated with an oxaliplatin-based regimen. *Journal of Clinical Oncology*.

[78] Garcia-Carbonero R., Rivera F., Maurel J. (2014). An open-label phase II study evaluating the safety and efficacy of ramucirumab combined with mFOLFOX-6 as first-line therapy for metastatic colorectal cancer. *Oncologist*.

[79] Tabernero J., Yoshino T., Cohn A. L. (2015). Ramucirumab versus placebo in combination with second-line FOLFIRI in patients with metastatic colorectal carcinoma that progressed during or after first-line therapy with bevacizumab, oxaliplatin, and a fluoropyrimidine (RAISE): a randomised, double-blind, multicentre, phase 3 study. *The Lancet Oncology*.

[80] Schwartzberg L. S., Rivera F., Karthaus M. (2014). PEAK: a randomized, multicenter phase II study of panitumumab plus modified fluorouracil, leucovorin, and oxaliplatin (mFOLFOX6) or bevacizumab plus mFOLFOX6 in patients with previously untreated, unresectable, wild-type KRAS exon 2 metastatic colorectal cancer. *Journal of Clinical Oncology*.

[81] Loupakis F., Cremolini C., Masi G. (2014). Initial therapy with folfoxiri and bevacizumab for metastatic colorectal cancer. *The New England Journal of Medicine*.

[82] Gruenberger T., Bridgewater J., Chau I. (2015). Bevacizumab plus mFOLFOX-6 or FOLFOXIRI in patients with initially unresectable liver metastases from colorectal cancer: the OLIVIA multinational randomised phase II trial. *Annals of Oncology*.

[83] Chibaudel B., Bonnetain F., Tournigand C. (2015). STRATEGIC-1: a multiple-lines, randomized, open-label GERCOR phase III study in patients with unresectable wild-type RAS metastatic colorectal cancer. *BMC Cancer*.

[84] Montagut C., Dalmases A., Bellosillo B. (2012). Identification of a mutation in the extracellular domain of the epidermal growth factor receptor conferring cetuximab resistance in colorectal cancer. *Nature Medicine*.

[85] Yonesaka K., Zejnullahu K., Okamoto I. (2011). Activation of ERBB2 signaling causes resistance to the EGFR-directed therapeutic antibody cetuximab. *Science Translational Medicine*.

[86] Bardelli A., Corso S., Bertotti A. (2013). Amplification of the MET receptor drives resistance to anti-EGFR therapies in colorectal cancer. *Cancer Discovery*.

[87] Bettegowda C., Sausen M., Leary R. J. (2014). Detection of circulating tumor DNA in early- and late-stage human malignancies. *Science Translational Medicine*.

[88] Wilhelm S. M., Dumas J., Adnane L. (2011). Regorafenib (BAY 73-4506): a new oral multikinase inhibitor of angiogenic, stromal and oncogenic receptor tyrosine kinases with potent preclinical antitumor activity. *International Journal of Cancer*.

[89] Grothey A., Van Cutsem E., Sobrero A. (2013). Regorafenib monotherapy for previously treated metastatic colorectal cancer (CORRECT): an international, multicentre, randomised, placebo-controlled, phase 3 trial. *The Lancet*.

[90] Li J., Qin S., Xu R. (2015). Regorafenib plus best supportive care versus placebo plus best supportive care in Asian patients with previously treated metastatic colorectal cancer (CONCUR): a randomised, double-blind, placebo-controlled, phase 3 trial. *The Lancet Oncology*.

[91] Santoro A., Comandone A., Rimassa L. (2008). A phase II randomized multicenter trial of gefitinib plus FOLFIRI and FOLFIRI alone in patients with metastatic colorectal cancer. *Annals of Oncology*.

[92] Tournigand C., Samson B., Scheithauer W. (2012). Bevacizumab (Bev) with or without erlotinib as maintenance therapy, following induction first-line chemotherapy plus Bev, in patients (pts) with metastatic colorectal cancer (mCRC): efficacy and safety results of the International GERCOR DREAM phase III trial. *Journal of Clinical Oncology*.

[93] Higgins B., Kolinsky K. D., Schostack K. (2011). Efficacy of vemurafenib (v), a selective v(600e)b-raf inhibitor, as monotherapy or in combination with erlotinib (erl) or erbitux (erb) and irinotecan (iri) doublets and triplets in a colorectal cancer (crc) xenograft model. *Journal of Clinical Oncology*.

[94] Yaeger R. D., Cercek A., O'Reilly E. M. (2015). Pilot study of vemurafenib and panitumumab combination therapy in patients with braf v600e mutated metastatic colorectal cancer. *Journal of Clinical Oncology*.

[95] Hochster H. S., Uboha N., Messersmith W. (2015). Phase II study of selumetinib (AZD6244, ARRY-142886) plus irinotecan as second-line therapy in patients with K-RAS mutated colorectal cancer. *Cancer Chemotherapy and Pharmacology*.

[96] Xu R. H., Shen L., Wang K. M. (2015). A randomized, double-blind, parallel-group, placebo-controlled, multicenter, phase ii clinical study of famitinib in the treatment of advanced metastatic colorectal cancer. *Journal of Clinical Oncology*.

[97] Asaoka Y., Ijichi H., Koike K. (2015). Pd-1 blockade in tumors with mismatch-repair deficiency. *The New England Journal of Medicine*.

[98] Köhne C.-H., Cunningham D., Di Costanzo F. (2002). Clinical determinants of survival in patients with 5-fluorouracil-based treatment for metastatic colorectal cancer: results of a multivariate analysis of 3825 patients. *Annals of Oncology*.

[99] Chibaudel B., Bonnetain F., Tournigand C. (2011). Simplified prognostic model in patients with oxaliplatin-based or irinotecan-based first-line chemotherapy for metastatic colorectal cancer: a GERCOR study. *Oncologist*.

[100] Kabbinavar F., Irl C., Zurlo A., Hurwitz H. (2008). Bevacizumab improves the overall and progression-free survival of patients with metastatic colorectal cancer treated with 5-fluorouracil-based regimens irrespective of baseline risk. *Oncology*.

[101] Desot E., de Mestier L., Volet J. (2013). Prognostic factors in patients with non resectable metastatic colorectal cancer in the era of targeted biotherapies: relevance of Köhne's risk classification. *Digestive and Liver Disease*.

[102] Sadanandam A., Lyssiotis C. A., Homicsko K. (2013). A colorectal cancer classification system that associates cellular phenotype and responses to therapy. *Nature Medicine*.

